# Coordination Reactions and Noncovalent Interactions of Polyamines with Nucleotides in Binary Systems and with Nucleotides and Copper(II) Ion in Ternary Systems

**DOI:** 10.1155/2010/740435

**Published:** 2010-09-05

**Authors:** Lechoslaw Lomozik, Anna Gasowska, Grzegorz Krzysko, Romualda Bregier-Jarzebowska

**Affiliations:** ^1^Faculty of Chemistry, A. Mickiewicz University, 60-780 Poznan, Poland; ^2^Faculty of Chemical Technology and Engineering, University of Technology and Life Sciences, 85-225 Bydgoszcz, Poland

## Abstract

Interactions of nucleotides (AMP, CMP) and 1,2-diaminopropane (tn-1) or 2-methyl-1,2-diaminopropane (tn-2) in metal-free systems as well as in the systems including copper(II) ions were studied. The composition and overall stability constants of the complexes formed were determined by the potentiometric method, whereas the interaction centres and coordination sites were identified by spectroscopic methods. It was found that phosphate groups of nucleotides and the protonated amine groups of polyamines are the centres of interaction. The differences in the interactions with the polyamines which act as models of biogenic amines are impacted by the presence of lateral chains (methylene groups) in tn-1 and tn-2. In the ternary systems with Cu(II) ions, the heteroligand complexes are mainly of the ML⋯L' type, in which the protonated polyamine is engaged in noncovalent interactions with the anchoring Cu(II)-nucleotide complex. The complexes formed in the Cu/NMP)/tn-1 system are more stable than those formed in the system with tn-2. The mode of coordination in the complex is realised mainly through the phosphate groups of the nucleotide with involvement of the endocyclic nitrogen atoms in a manner which depends upon the steric conditions and in particular on the number of the methylene groups in the polyamine molecule.

## 1. Introduction

Polyamines (PA) represent an important group of molecules present in practically all living organisms. The concentrations of polyamines depend upon the type and age of cells. Particularly high levels of PA have been observed in young cells (including neoplasmic cells) [1–6] and this information can be used in clinical diagnostics and treatment monitoring [7, 8]. Biogenic amines take part in many important biological processes, including growth or death of cells, stabilisation of membranes, and stabilisation and replication of nucleic acids or proteins [9–11]. Aliphatic amines containing protonated amine groups at physiological pH can interact with the negatively charged fragments of other biomolecules, for example, amino acids, enzymes, DNA, or RNA [5, 12–17]. With respect to nucleic acids, these interactions can lead to structural changes at various levels of organisation and illustrate the role of PA in the process of genetic information transfer [18]. The PA-DNA interaction leads to stabilisation of the helix, resulting in increases in the DNA melting point by as much as 40°C [19, 20]. The characteristics of the interactions of the polyamine with the other biomolecules vary in a manner which depends upon the amine chain length and the number of amine groups [21]. As suggested by the polyelectrolytic theory proposed by Manning [22, 23] the efficiency of the interactions can be determined by the charge of the PA molecule. However, an important role for PA as a structure-influencing factor has also been indicated [19, 24–26]. Therefore, in addition to the electrostatic forces, steric effects [27–30] should be considered. Spermine is the longest biogenic amine. This molecule has been found to be more effective than Put (putrescine) and Spd (spermidine) in inducing structural changes in nucleic acid molecules [27, 31, 32]. Since the mechanisms of many processes have not yet been fully explained, attempts should be made to determine the influence of the number and types of donor ligands and the presence of side chains (steric hindrance) on the characteristics of the reactions. On the other hand, it is known that the characteristics of the interactions also depend upon the presence of metal ions. Coordination sites for bio-ligands may simultaneously function as potential centres for noncovalent interactions. Metal ions in living organisms should be considered as interfering agents which compete with polyamines in reactions with nucleic acid fragments [33–36].

This paper describes the interactions occurring in binary and ternary systems of 1,2*-*diaminopropane (tn-1) and 1,2-diamino-2-methylopropane (tn-2) with nucleotides (AMP, CMP) and copper(II) ions. 

## 2. Experimental

Copper(II) nitrate was purchased from POCH Gliwice (Poland) and used after recrystallising it twice from H_2_O. The method of determining the concentration of Cu(II) in the parent solution was performed as previously described in [36, 37]. Adenosine 5′-monophosphate sodium salt (AMP) and cytidine 5′-monophosphate (CMP) were purchased from Sigma, and dihydrochloride 1,2-diaminopropane (tn-1) was purchased from Aldrich and used without additional purification. 1,2-diamino-2-methylpropane (tn-2) was purchased from Sigma. The tn-2·2HCl ligand was prepared by dissolving an appropriate amount of free tn-2 followed by addition of an equimolar amount of HCl. The white precipitate was recrystallised, washed with methanol and dried in a desiccator over P_4_O_10_. Results of elemental analyses (%C, %N, and %H) performed on the Elemental Analyzer CHN 2400, Perkin-Elmer were consistent with the theoretically calculated values (±0.5%).

A potentiometric titration was performed using a Methrom system (713 pH-meter, 725 dosimat, 728 stirrer and glass electrode 6.233.100). The electrode was calibrated in terms of hydrogen ion concentration [38]. In the binary metal-free systems, the concentrations of AMP, CMP, and tn-1 and tn-2 were each 0.01 M, while in the systems containing Cu(II) ions, the Cu(II) concentration was between 1 × 10^−3^ M to 1.5 × 10^−3^ M (the ratio of the metal to ligand concentrations in the systems with Cu(II) varied from 1 : 3.3 to 1 : 4.6). The measurements were performed under a neutral gas atmosphere at an ionic strength of *μ* = 0.1 (KNO_3_) and a temperature of 20 ± 1°C, with a CO_2_-free solution of NaOH as a titrant. The titration was conducted over a pH range from 2.5 to about 10.5. At least 5 titrations were performed with 150 to 350 points for each titration. The model used to describe the formation of the complexes was selected with the help of the SUPERQUAD program using data obtained from potentiometric experiments (taking into account only the part of the titration curve corresponding to points where the system was free of precipitate) [39]. 

SUPERQUAD uses the nonlinear method of least squares to minimize the sum (*S*) of the squares of residuals between the observed quantities (*f* ^obs^) and those calculated on the basis of the model (*f* ^calc^)

(1) $$S=\overset{n}{\underset{i=1}{\sum }}w_{i}{\left(f^{\text{obs}}-f^{\text{calc}}\right)}^{2},$$

where *n* is the number of measurements and *w*_*i*_ is the statistical weight. 

The iteration procedure allows determination of the types of complexes (stoichiometry) and the thermodynamic stability of the complexes formed in the binary and ternary systems [39].

(2) $$\begin{array}{c} n\text{M}\;+\;p\text{L}\;+\;q{\text{L}}^{\prime }+\;r{\text{H}}^{+}⇆{\text{M}}_{n}{\text{L}}_{p}{\text{L}}_{q}^{\prime }{\text{H}}_{r}\\ \beta =\frac{\left[{\text{M}}_{n}{\text{L}}_{p}{\text{L}}_{q}^{\prime }{\text{H}}_{r}\right]}{{\left[\text{M}\right]}^{n}{\left[\text{L}\right]}^{p}{\left[{\text{L}}^{\prime }\right]}^{q}{\left[\text{H}\right]}^{r}}. \end{array}$$

The testing began with the simplest hypothesis and then in subsequent steps, the models were expanded to include additional species. The results were scrutinized to eliminate the species rejected by the refinement processes. The assumed model was verified by analysis of the statistical parameters as well as the convergence of the experimental and simulated curves. The criteria of the correct model verification have been described in earlier publications [40, 41]. The distribution of particular species was obtained using the HALTFALL program [42]. The samples used for the NMR experiments were prepared by dissolving AMP, CMP, tn-1, tn-2, and Cu(II) in D_2_O and adjusting the pD by addition of NaOD or DCl. The pD value was corrected according to the formula pD = pH (meter readings) + 0.4 [43]. The concentration of the ligands in the samples was 0.01 M, and the ratio of the Cu(II) to ligand was varied from 1 : 100 to 1 : 200. The ^13^C NMR spectra were obtained using a Gemini 300 VT Varian NMR spectrometer using dioxane as an internal standard. The positions of signals in the ^13^C NMR spectra are expressed with respect to tetramethylsilane (TMS). Measurements of ^31^P NMR were made using a Unity-300 Varian NMR spectrometer (with H_3_PO_4_ as a standard). The UV-Vis spectra were obtained using a JASCO V-500 UV-Vis spectrophotometer for ligand concentrations similar to those of the samples used for potentiometric titrations, with a metal-to-ligand ratio of 1 : 3.5. EPR measurements were obtained in a 3 : 1 water:glycol mixture at 77 K on an SE/X 2547 spectrometer G9 (Radiopan).

## 3. Results and Discussion

**Figure 1:**
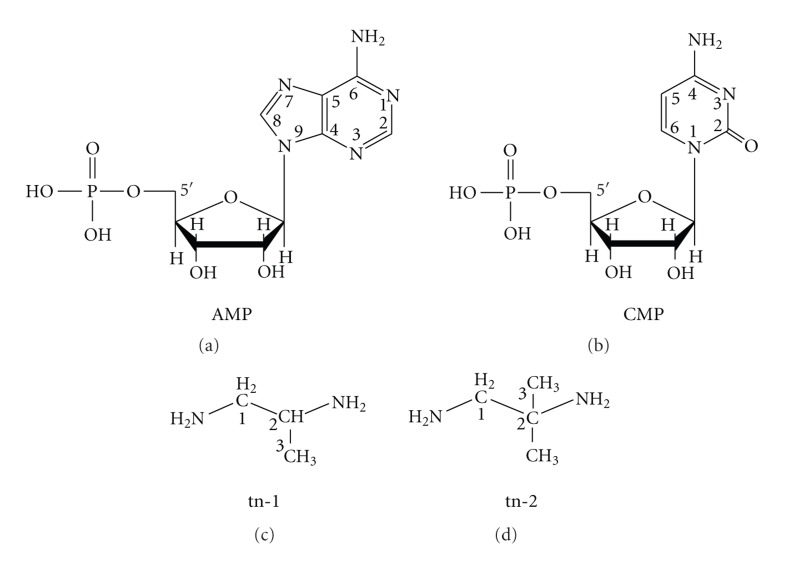
Chemical formulae of the bioligands studied.

The ligands are discussed with respect to the atom numbering scheme shown in Figure 1.

### 3.1. Equilibrium and Spectral Studies of NMP/tn-1 Systems (NMP = AMP or CMP)

For the metal-free systems, reactions between adenosine-5′-monophosphate (AMP) or cytidine-5′-monophosphate (CMP) and 1,2-diaminopropane (tn-1) were observed in pH ranges where the partly deprotonated nucleotide acts as a negative centre and the protonated polyamine acts as a positive centre in ion-ion or ion-dipole interactions as recently determined for similar systems [15, 16, 21, 44–47]. This interaction leads to formation of molecular complexes which can be described by

(3) $$\begin{array}{c} {\text{H}}_{x}\left(\text{NMP}\right)+\;{\text{H}}_{y}\text{PA}⇆{\left(\text{NMP}\right)}_{\text{H}(x+y-n)}\left(\text{PA}\right)+\;n{\text{H}}^{+}. \end{array}$$

**Figure 2:**
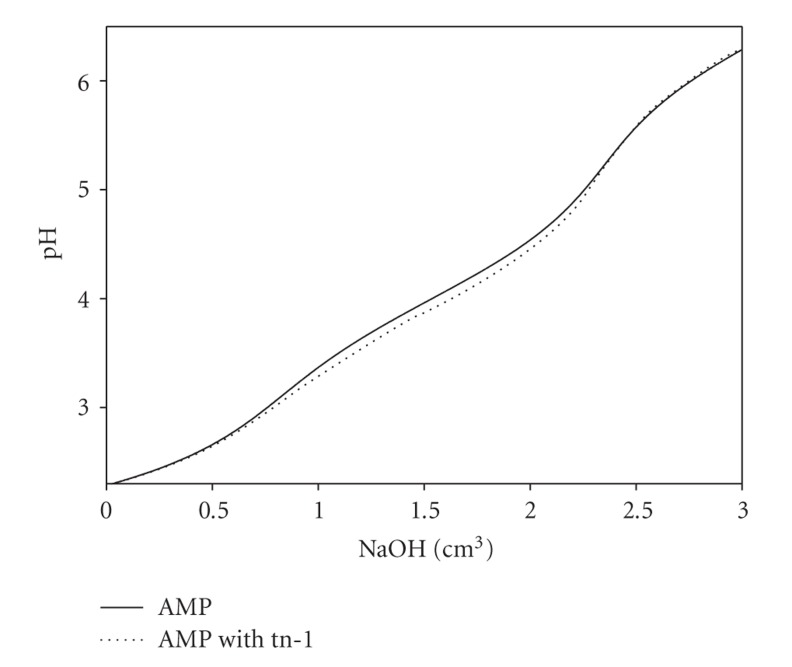
Titration curves of the AMP and AMP/tn-1 systems; C_AMP_ = 0.01 M, C_tn-1_ = 0.01 M C_NaOH_ = 0.1915 M

The release of protons permits the use of the potentiometric method used in this study. The difference between the titration curve of the nucleotide in the absence and in the presence of a diamine confirms that proton dissociation occurs as a result of the substrate interaction (Figure 2).

**Figure 3:**
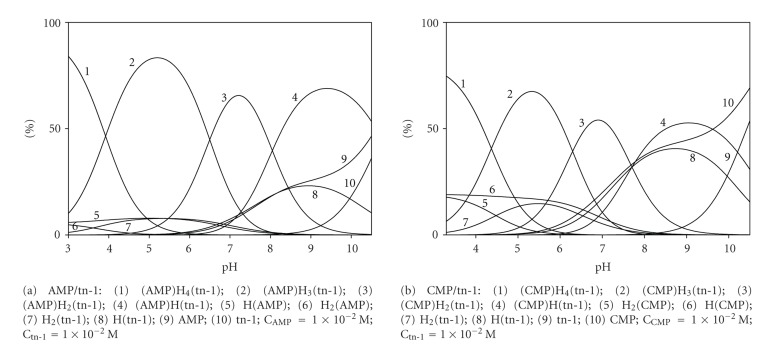
Distribution diagrams for the AMP/tn-1 and CMP/tn-1 systems; percentage of the species refers to total ligands.

In the pH range from 2.5 to 10.5, several species of (NMP)H_*x*_(tn-1) occur, where *x* = 4, 3, 2, and 1. Relative concentrations and pH ranges for the complex formation are given in Figure 3.

In the complexes of both nucleotides with tn-1, the tetraprotonated species (NMP)H_4_(tn-1) is formed within a pH range extending to about pH 6. The stability constants and equilibrium constants of adduct formation (together with the protonation constants of both diamines) are shown in Table 1. In the pH range investigated, the protonation constants of tn-1 (a compound of high basicity) indicates that both amine groups from this ligand are protonated and interact as positive centres with the partly deprotonated phosphate groups of the nucleotide which act as negative centres. This is confirmed by the results of the spectroscopic studies discussed below.

Because of different stoichiometric compositions of particular species, the overall stability constants log *β* cannot be directly applied in analysis of the characteristics of the interactions. Therefore, the efficiency of bonding was estimated on the basis of the equilibrium constants calculated for species such as (AMP)H_4_(tn-1), where log *K*_*e*(AMP)H_4_(tn-1)_ = log *β*_(AMP)H_4_(tn-1)_ − log *β*_(H_2_AMP)_ − log *β*_(H_2_tn-1)_ = 4.49. Similar calculations were performed for the complexes formed in the Cu/NMP/tn-1 and Cu/NMP/tn-2 ternary systems. 

Higher values of the constant of adduct formation were determined for the systems containing purine nucleotide (AMP) relative to those of the systems containing pyrimidine nucleotide (CMP) as indicated in Table 1. This suggests that the differences in the noncovalent interactions are a result of different numbers of endocyclic nitrogen atoms. On the other hand, a series of tetra-, tri-, and diprotonated complexes of AMP or CMP with tn-1 have similar values of log *K*_*e*_. This indicates that the mode of interactions is the same despite the increased degree of deprotonation of tn-1 and the nucleotide.

(NMP)H_3_(tn-1) complexes dominate at a pH of about 5.5, while (NMP)H_2_(tn-1) complexes exist near physiological pH (~7) where binding of the AMP or CMP systems occupies about 90% and 75% of the ligand, respectively. A distinct decrease in log *K*_*e*_ is observed for the monoprotonated adduct which is dominant in the range from pH 8 to pH 10. This is undoubtedly a consequence of the disappearance of positively charged centres with total deprotonation of one of the –NH_3_^+^ groups of the amine and partial deprotonation of the other group, as follows from the protonation constant values of tn-1 (Table 1). The decreased number of positive centres at higher pH values restricts the possibility of interactions with the two endocyclic nitrogen atoms of the purine bases. The restriction of interactions is confirmed by the observation of a decrease in the difference of the formation constants (log *K*_*e*_) of the adducts (AMP)H_*x*_(tn-1) relative to the (CMP)H_*x*_(tn-1) adducts (where *x* = 4, 3, 2, and 1, the differences are 0.58 > 0.56 > 0.37 > 0.21, resp.) Table 1. Total deprotonation of the amine leads to disappearance of the molecular complexes at high pH. The above results also confirm the conclusion drawn from our earlier investigations that at least two centres of interaction are necessary to obtain a relatively stable adduct [16, 21, 48].

The shifts of NMR signals caused by changes in the electron density on the atoms located near the reaction centres permit identification of the sites of noncovalent interactions. In the pH range where the (AMP)H_4_(tn-1) complex dominates, the sites are the phosphate groups from the nucleotide and the two protonated amine groups from tn-1, as indicated by the chemical shifts of ^13^C and ^31^P NMR presented in Table 2.

 In the ^13^C NMR spectrum, the changes in the positions of the signals assigned to C(5) and C(8) atoms of the purine ring of AMP are 0.075 and 0.049 ppm, respectively. Although these changes are small, they are still much greater than those of the other carbon atoms of the base. This indicates that the main reaction centre for the negative charge is N(7). When the pH increases and the (AMP)H_3_(tn-1) species is formed, the strength of the interaction at N(1) increases as proven by the increasing changes in the chemical shifts of the ^13^C NMR signals assigned to the carbon atoms which are located close to the nitrogen atom. For example, the chemical shift of C(2) increases by 0.013 ppm from pH 3 to pH 5, and the chemical shift of C(6) increases by 0.016 ppm from pH 3 to pH 5. The ((AMP)H_4_(tn-1)) complex dominates at pH 3 and the ((AMP)H_3_(tn-1)) complex dominates at pH 5 as indicated in Table 2. Changes in the chemical shift of C(4), which is located far from the nitrogen atoms that could act as reaction sites, are insignificant across the entire pH range studied. Significant changes in the positions of signals in the ^31^P NMR spectra (Table 2) provide evidence of involvement of the phosphate group in the formation of the (AMP)H_3_(tn-1) adduct. The positive reaction centres are the protonated amine groups. 

In the (AMP)H_2_(tn-1) complex, the strength of the interactions decrease between the AMP phosphate group and one of the amine groups of tn-1. This is deduced from the changes in the chemical shifts of C_1_, C_2_, and C_3_ of tn-1 (Table 2). This decrease is correlated with the acid-base character of the amine. In the pH range where (AMP)H_2_(tn-1) dominates, one of the amine groups of tn-1 begins to deprotonate, and its positive charge decreases. Relative to the situation observed for biogenic amines where both –NH_3_^+^ groups are involved in the interactions [14, 15, 47], the involvement of only one of these groups is not only a consequence of a lower charge but also the presence of steric hindrance provided by the side chain of the tn-1 molecule.

In the (AMP)H(tn-1) adduct, the phosphate group and the endocyclic nitrogen atoms from the purine base interact with one amino group of tn-1. The amino group at C_2_ of diamine does not participate in the formation of the adduct. This conclusion is drawn from the results of spectral studies performed at pH 9 which are well correlated with the results of the equilibrium studies where a much smaller log *K*_*e*_ is observed for the monoprotonated complex as indicated in Table 1. Relatively large changes in the chemical shifts in the ^31^P NMR spectra of the AMP phosphate group are attributed to the noncovalent interactions and prove that the phosphate group is involved in efficient formation of all adducts. 

The pattern of changes observed for the chemical shifts in the spectra of the complexes formed by the CMP/tn-1 system indicates that the mean negative centre of interaction is the phosphate group of the nucleotide (Table 2). The endocyclic nitrogen atom of the pyrimidine ring is not a reaction centre. This was confirmed by the analysis of the changes in the chemical shifts. One exception is noted for involvement of the endocyclic nitrogen atom in formation of the (CMP)H_3_(tn-1) adduct. Total deprotonation of the phosphate group of CMP (log *K* = 6.42) causes an increase in the efficiency of formation of (CMP)H_2_(tn-1) which appears at higher pH values. This total deprotonation also causes a higher log *K_e_* value as indicated in Table 1. 

The equilibrium constants for formation of CMP complexes are lower than for AMP complexes (Table 1). In contrast to the AMP/tn-1 system, all (CMP)/(tn-1) complexes which have both diamine amino groups in the protonated state, are involved in the interactions. One exception is the (CMP)H(tn-1) complex, as indicated by the analysis of changes in the chemical shifts of C_1_, C_2_, and C_3_ (Table 2). The observation of small but significant changes in the chemical shifts of the C_2_ and C_3_ atoms of the monoprotonated species of tn-1 can be explained by the fact that the endocyclic nitrogen atom does not take part in the interactions. In contrast, the analogous AMP species must have an effect on the solution structure of the complex and the character of the through-space interactions. 

The (CMP)H(tn-1) complex dominates at a pH of about 9. Deprotonation of one amine group causes a decrease in the efficiency of interaction with phosphate group of CMP. This causes a reduction in the value of log *K*_*e*_ as indicated in Table 1.

### 3.2. Equilibrium and Spectral Studies of NMP/tn-2 (NMP = AMP or CMP) Systems

The 1,2-diamino-2-methylopropane (tn-2) interactions with AMP and CMP over a pH range from 2.5 to 10.5 produce a series of molecular complexes according to the formula (NMP)H_x_(tn-2) where *x* = 4, 3, 2, and 1. The protonation constants of the amine (log *K*_1_ = 9.80, log *K*_2_ = 6.61) prove that in the (NMP)H_4_(tn-2) complexes which are formed at low pH, both amino groups of tn-2 are protonated and interact as positive reaction centres with the negative centres of the nucleotide. Complexes (NMP)H_3_(tn-2) and (NMP)H_2_(tn-2) dominate at pH 5.5 and 7, respectively. The log *K*_*e*_ values of (AMP)H_3_(tn-2) and (AMP)H_2_(tn-2) are 3.92 and 3.99, respectively. The difference between these values is considered insignificant. This suggests that similar interaction modes are involved. A similar trend is observed for the analogous CMP complexes (the equilibrium constants of formation of tri- and diprotonated complexes are 3.55 and 3.78, resp.). Significant differences between the constants of formation are noted between (NMP)H_2_(tn-2) and (NMP)H(tn-2) as shown in Table 1. In the pH range of domination of the latter species (pH 9), one of the amino groups of tn-2 is significantly deprotonated (log *K*_1_ = 6.61). This reduces the extent of positive charge at the amino group and leads to reduction of the stability of the adduct. This observation is in good agreement with the conclusion that in the (NMP)H_*x*_(tn-2) adducts where *x* = 4, 3, and 2, both amino groups provide positively charged reaction centres. 

**Figure 4:**
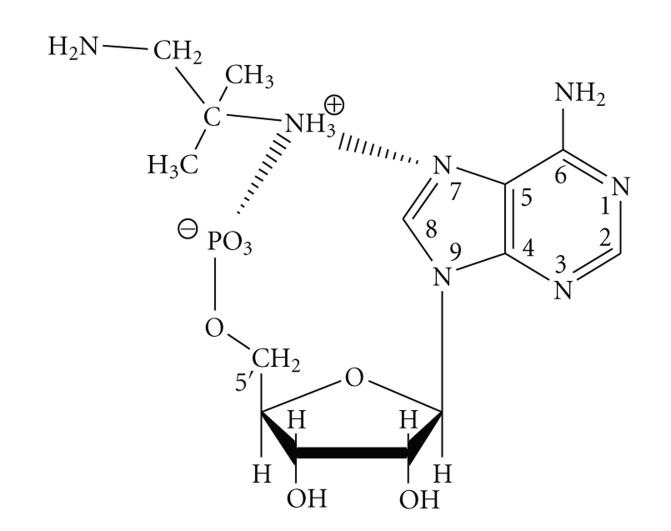
Tentative mode of interaction in the (AMP)H(tn-2) complex.

The effect of steric hindrance on the characteristics of the interactions is illustrated by a comparison of the log *K*_*e*_ values of tn-1 and tn-2 complexes. This indicates that the stability of the adduct of the former ligand is almost always significantly greater than the stability of the latter (Table 1). The differences in the stability of these adducts are not related to the charge states of tn-1 and tn-2 because the range of deprotonation of tn-1 and tn-2 is the same, as indicated by their similar protonation constants. Therefore, it is mainly the presence of the second methyl group, which alters the steric conditions, that has an effect on the character of the adducts. When only one of the amino groups takes part in the noncovalent interactions, the presence of another substituent does not significantly hinder the formation of the adduct (Figure 4).

In the pH range where the (AMP)H_4_(tn-2) complex is dominant, the analysis of the changes in the NMR signal positions corresponding to atoms implicated in the noncovalent interactions provides an indication that the phosphate group is the negative centre. The NMR spectra indicate small but systematic changes in the chemical shifts of C(5′) and considerable (in view of the type of interactions) changes in the chemical shifts of phosphorus atoms in the ^31^P NMR spectra. On the other hand, the change in the chemical shift of C(6) at pH 3 is only 0.017 ppm (Table 2). This indicates that the endocyclic nitrogen atom of the purine ring is excluded from participation in weak interactions. Of course at such low pH values, the amino groups are fully protonated and act as positive centres of reaction as confirmed by the changes in the chemical shift of the carbon atoms from tn-2. According to our previous investigations, the magnitude of the changes is affected by the type of interactions and the type of ligand [21, 48].

At pH values near 5 where (AMP)H_3_(tn-2) dominates, the endocyclic nitrogen atom of the purine ring is deprotonated (log *K* for AMP is 4.02), and this nitrogen atom functions as an additional centre of interaction as indicated by the considerable changes observed in the NMR spectra (Table 2). However, no increase in the equilibrium constant is noted when comparing the values obtained for the tetra- and triprotonated complexes. This is probably related to the steric differences of the substrates of both adducts. The changes in the NMR spectrum of (AMP)H_2_(tn-2) and the fact that the log *K*_*e*_ value is similar to that of the triprotonated adduct suggests that the phosphate group and the endocyclic nitrogen atoms are the negative reaction centres for the diprotonated complex. The pattern of changes observed for the signals assigned to the carbon atoms from tn-2 indicates that there is a lesser extent of engagement of the amino group at the carbon atom to which two methyl groups are attached. This is surely a result of steric hindrance but there may also be a contribution from the partial deprotonation of the amino group at about pH 7 (log *K*_1_ of tn-2 is 6.61) where the (AMP)H_2_(tn-2) complex dominates. The effect of deprotonation of the tn-2 amino group is significant for (AMP)H(tn-2), in which only the –NH_3_^+^ at C_1_ acts as a positive reaction centre. The changes in the chemical shifts assigned to the C_1_, C_2_, and C_3_ atoms from tn-2 (0.107, 0.006, and 0.017 ppm, resp.) confirm this conclusion. The NMR data indicating that only one of the amino groups of tn-2 is involved in the formation of the adduct is in agreement with the results of the potentiometric study. The formation constant (log *K*_*e*_) of the monoprotonated complex is much lower than those of the other species as indicated in Table 1. 

In all of the (CMP)H_4-1_(tn-2) complexes, the phosphate group of the nucleotide participates, but there is no involvement of the endocyclic nitrogen atom of pyrimidine ring. This is established on the basis of the NMR results (Table 2). Despite its deprotonated state, N(3) is not involved in the interactions. The changes in the chemical shifts of C(2) and C(4) in the NMR spectra measured at pH 7 are only 0.018 0.029 and at pH 9 only 0.003 and 0.036 ppm. The lack of engagement of N(3) is also indirectly confirmed by the observation that the CMP adducts have lower stability than the AMP adducts (Table 2). Despite the relatively small changes in the chemical shifts of the carbon atoms of tn-2, the pattern of their changes suggests the participation of the two amino groups in the interactions (Table 2), except for the (CMP)H(tn-2) adduct which has one positive interaction center. This conclusion is supported by the clear decrease observed for the log *K*_*e*_ of this species relative to the corresponding values of the adducts (CMP)H_*x*_(tn-2), *x* = 4, 3, and 2, Table 1).

### 3.3. Equilibrium and Spectral Studies of Cu/NMP/tn-1 (NMP = AMP or CMP) Ternary Systems

**Figure 5:**
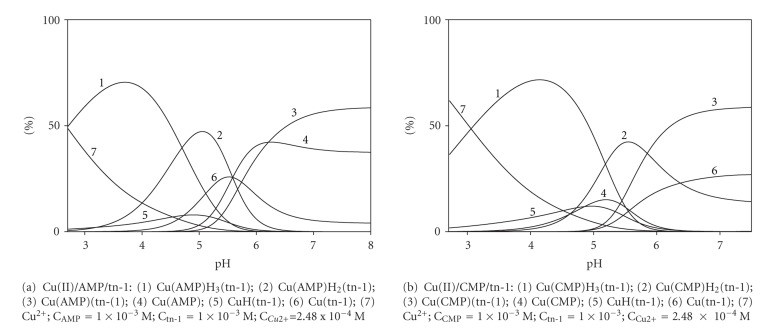
Distribution diagrams for the Cu(II)/AMP/tn-1 and Cu(II)/CMP/tn-1 systems; percentage of the species refers to total metal.

According to computer analysis of the potentiometric data, in the presence of Cu(II) the combinations of tn-1 with AMP or CMP form the following heteroligand complexes: Cu(AMP)H_3_(tn-1), Cu(AMP)H_2_(tn-1), Cu(AMP)(tn-1), Cu(CMP)H_3_(tn-1), Cu(CMP)H_2_(tn-1), and Cu(CMP)(tn-1). Interestingly, no monoprotonated species are detectable. Concentrations of the specific complexes are shown in Figure 5, and the stability constants and the equilibrium constants of formation are shown in Table 3. The upper limit of the pH range is pH 8. Precipitates form at higher pH values.

The Cu(NMP)H_3_(tn-1) complexes are dominant at about pH 4, while Cu(NMP)H_2_(tn-1) dominates from pH 5 to 6. The presence of three labile protons in Cu(AMP)H_3_(tn-1) and Cu(CMP)H_3_(tn-1) suggests that these triprotonated species are molecular complexes of the ML⋯L' type. The copper ions become coordinated to the nucleotide and the fully protonated tn-1 (which has high protonation constants as indicated in Table 1) is engaged in noncovalent interactions with the anchoring complex. This model is in agreement with the conclusions drawn from the spectral studies. The energies of the d-d transitions at pH 4, in the pH range of domination of the triprotonated complex are 802 and 769.5 nm for Cu(AMP)H_3_(tn-1) and Cu(CMP)H_3_(tn-1), respectively. This indicates that coordination occurs only through the oxygen atoms of the phosphate group, as deduced from a comparison of the values of analogous systems in previous investigations [14–16, 49, 50]. The formation of the {O_*x*_} chromophore type is also indicated by EPR parameters g_||_ = 2.367 and 2.332, as indicated in Table 4. Thus, the results of UV-Vis and EPR spectroscopic studies clearly confirm formation of an adduct with an intermolecular interaction between the anchoring CuH(AMP) complex and the protonated amine H_2_(tn-1).

This interpretation is supported by the results of the NMR investigation. The chemical shifts of the relevant signals in the ^13^C and ^31^P NMR spectra originating from the ligand in the complex relative to those of the free ligand were interpreted by taking into account the limitations which arise from the presence of paramagnetic ions [16, 44, 51, 52]. The chemical shifts of the ligands were obtained from the literature and from our recent study [16]. In order to minimize the NMR signal broadening, the spectra were recorded with very low concentrations of the metal. Analysis of the distribution diagrams has shown that differences in the concentrations of the substrate have no considerable influence on the ranges of complex domination. This has also been recently observed in similar systems [15, 53]. Of course, the concentration of a particular species depends upon the M : L ratio. Moreover, only good correlations between NMR results and those obtained from independent spectral methods allow us to draw a conclusion about an interaction mode. 

The change in the chemical shift of the signal from C(5′) (located near the phosphate group) in the ^13^C NMR spectrum of Cu/AMP/tn-1 at pH 4 by 0.325 ppm and the change in the chemical shift of the phosphorus atom in the ^31^P NMR spectrum by 0.418 ppm confirm the participation of the phosphate group in the coordination. It should be noted that the 0.037 ppm change in the chemical shift of C(4) of ATP, which is located far from the reaction centre, is much smaller.

The UV-vis and EPR spectra which indicate the formation of the {O_*x*_} chromophore indicate that the endocyclic nitrogen atoms from AMP are not involved in the process of coordination of the metal. The changes in the chemical shifts originate from C(2), C(6), C(5), and C(8) atoms in the ^13^C NMR spectra (C(2) = 0.253, C(6) = 0.266, C(5) = 0.378, and C(8) =0.332 ppm). These data indicate that the listed carbon atoms participate in noncovalent interactions as negative centres. Moreover, small but systematic changes in the chemical shifts arising from the carbon atoms of tn-1 (relative to the signal positions in the spectrum of the free ligand) were observed. The fully protonated -NH_3_^+^ groups provide the positive interaction centres at low pH. The magnitude of the changes in the chemical shifts is typical for this type of interaction [14–16, 51]. 

The spectral parameters of the Cu(CMP)H_3_(tn-1) complex (C(5′) 0.137 ppm, ^31^P NMR 0.252 ppm) indicate that the only sites involved in binding metal ions are the oxygen atoms from the phosphate group ({O_*x*_} chromophore). The endocyclic N(3) atoms are negative centres involved in weak interactions. The protonated polyamine –NH_3_^+^ groups act as a positive entity as confirmed by the changes in the chemical shifts arising from C_1_, C_2_, and C_3_ (0.067, 0.052, and 0.047 ppm, resp.). These observations are similar to those observed for the Cu(AMP)H_3_(tn-1) adduct. Analogous modes of interaction of the triprotonated complexes of both nucleotides are confirmed by their similar stability constants (Table 3). A potentially controversial interpretation is that there is a significant difference in the energy of the d-d transitions of the Cu(AMP)H_3_(tn-1) complexes and the Cu(CMP)H_3_(tn-1) complexes which have *λ*_max _ = 802 and 769.5, respectively. Although there is no other evidence, the above difference suggests the participation of the phosphate group of CMP in both metal coordination and noncovalent interactions. This dual participation of a phosphate group has been recently observed in a ternary system with another pyrimidine nucleotide; uridine 5′-monophosphate [46, 54]. 

**Figure 6:**
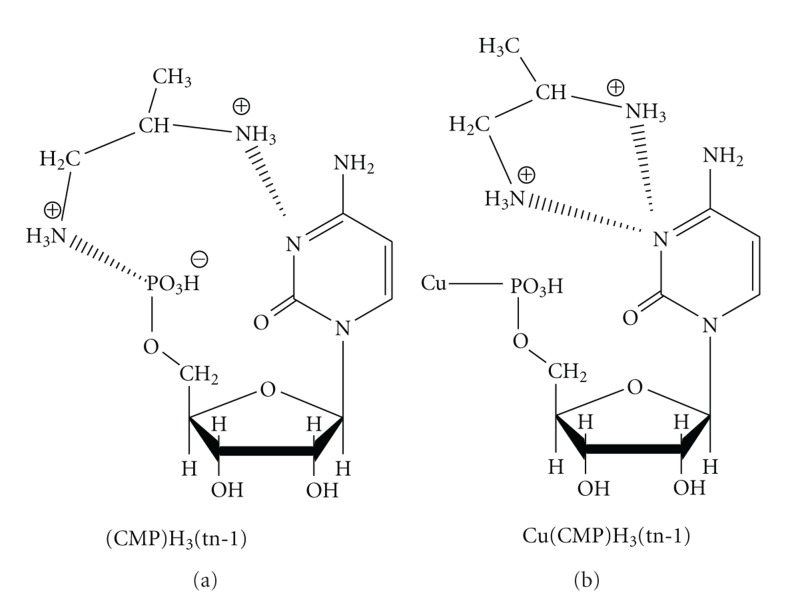
Tentative mode of interaction in (CMP)H_3_(tn-1) and Cu(CMP)H_3_(tn-1) complexes.

Introduction of metal ions into the CMP/tn-1 system changes the mode of interactions discussed above for the metal-free adduct of (CMP)H_3_(tn-1) as schematically shown in Figure 6.

In the Cu(NMP)H_2_(tn-1) complexes which dominate at pH 5, the UV-Vis and EPR data (*λ*_max_ = 697.5 nm or 702 nm and g_||_ = 2.243 or 2.238) for the systems with AMP and CMP, respectively, indicate the formation of a {1N,O_*x*_} chromophore. The change in the type of coordination relative to the triprotonated complex is undoubtedly a consequence of deprotonation of the endocyclic nitrogen atom. The pattern of changes in the NMR spectra supports the interpretations made on the basis of the EPR and UV-Vis data. For example, the changes in the chemical shifts originating from C(5*'*) and phosphorus of AMP as a result of coordination are 0.092 and 0.147 ppm, while the changes in the chemical shifts of C(2), C(6), C(5), and C(8) which are located relatively close to the endocyclic nitrogen atoms are 0.102, 0.097, 0.121, and 0.111 ppm, respectively. In contrast, the change in the chemical shift for C(4), which is located far from the coordination site is only 0.017 ppm. It is likely that the donor atom is N(7). Coordination through N(1) is unlikely because it would require a conformational change from the typical anticonformation to the energetically unfavourable syn conformation. The pH range for formation of the Cu(NMP)H_2_(tn-1) complex and the protonation constants of this complex suggest that the two amine groups of tn-1 are protonated. The type of chromophore deduced from EPR and UV-Vis spectra and the fact that changes are also observed in the chemical shifts of the amine carbon atoms (with values typical of the interactions of this type established in the studies of analogous systems [15, 16]) suggest that the protonated groups of tn-1 represent positive reaction centres for noncovalent interactions with the anchoring Cu(AMP) or Cu(CMP) complexes. The presence of only one positive centre in the amine molecule indicates that if the monoprotonated complex forms at all, it occurs at undetectably low levels. 

The values of *λ*_max_ and g_||_ for Cu(AMP)(tn-1) and Cu(CMP)(tn-1) adducts measured at pH 7 are *λ*_max_ = 660 nm, g_||_ = 2.251 and *λ*_max_ = 633 nm, g_||_ = 2.263, respectively. These data support the existence of the {2N,O_*x*_} coordination type. At pH 7, insignificant changes are observed for the ^13^C NMR signals assigned to the carbon atoms in the vicinity of the endocyclic nitrogen atoms (AMP: C(2) = 0.013, 0.007, C(6) = 0.005 and C(8) = 0.009 ppm and CMP: C(2) = 0.009 and C(4) = 0.003 ppm). These observations indicate that these carbon atoms are not involved in coordinating interactions at pH 7. Therefore, two donor nitrogen atoms must be provided by the tn-1 molecule. Evidence for this is provided by the changes in the chemical shifts assigned to carbon atoms located close to the nitrogen atoms of the amine. Moreover, the equilibrium constants for formation of Cu(AMP)(tn-1) and Cu(CMP)(tn-1) are higher than the equilibrium constants of formation of the binary Cu(tn-1) complex with monofunctional coordination [55]. These observations indicate that both nitrogen atoms of tn-1 are involved in metal coordination. Moreover, the significantly higher values of logK_e_ for Cu(AMP)(tn-1) and Cu(CMP)(tn-1) relative to Cu(AMP)H_2_(tn-1) and Cu(CMP)H_2_(tn-1), (Table 3) confirm the difference in the interaction mode for both species and indicate formation of a Cu–N bond (from tn-1).

### 3.4. Equilibrium and Spectral Studies of Cu/NMP/tn-2 (NMP = AMP or CMP) Ternary Systems

**Figure 7:**
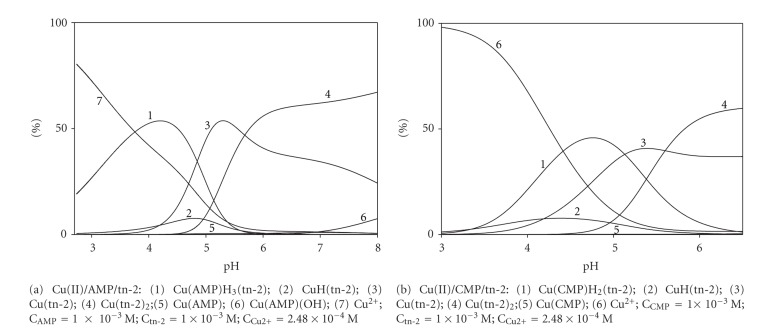
Distribution diagrams for the Cu(II)/AMP/tn-2 and Cu(II)/CMP/tn-2 systems; percentage of the species refers to total metal.

The coordination character of 1,2-diamino-2-methylopropane (tn-2) differs significantly from that of tn-1. Computer analysis of the potentiometric data has shown that only the heteroligand Cu(AMP)H_3_(tn-2) complex is formed and reaches a maximum concentration at pH 4.5 (Figure 7).

**Figure 8:**
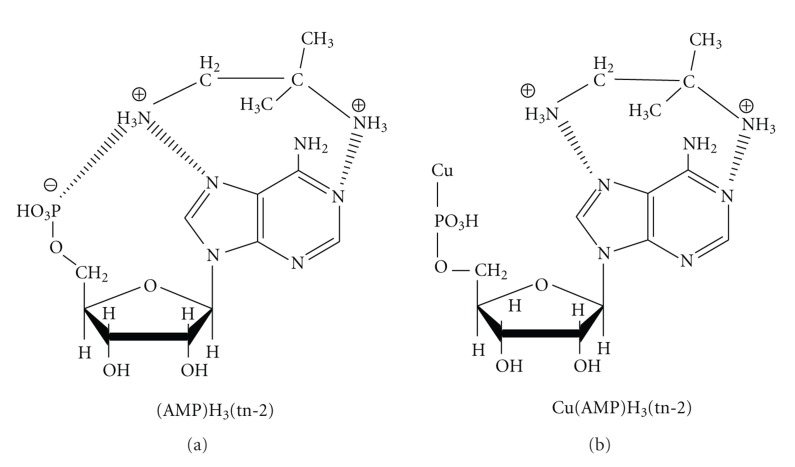
Tentative mode of interaction in (AMP)H_3_(tn-2) and Cu(AMP)H_3_(tn-2) complexes.

The spectroscopic parameters (*λ*_max_ = 763 nm and g_||_ = 2.364) indicate the formation of a chromophore of the {O_*x*_} type with coordination by oxygen atoms of the phosphate group, as confirmed by changes in the NMR signals assigned to C(5′) and the phosphate atom of 0.344 and 0.404 ppm, respectively. Taking into account the fact that the metal ion coordination occurs only through the –PO_3_^−^ group of AMP, the changes in the NMR signals assigned to the carbon atoms in the vicinity of the endocyclic nitrogen atoms from the purine ring (C(2) = 0.918 ppm, C(6) = 0.455 ppm, C(5) = 0.421 ppm, and C(8) = 0.584 ppm), suggest that N(1) and N(7) which have high electron density act as negative centres of interactions. The number of hydrogen atoms in the adduct and the protonation constants of tn-2 clearly show that both amine groups are protonated and act as the positive centres in the noncovalent interactions leading to formation of the ML ⋯ L adduct. As follows from the changes in the chemical shifts of C_1_, C_2_, and C_3_ of the amine equal to 0.000, 0.094, and 0.046 ppm, only the –NH_3_^+^ group at C_2_ is involved in the noncovalent interactions. The presence of the metal ions induces changes in the mode of interaction relative to that in the metal-free system of AMP/tn-2. The coordination of Cu(II) by the phosphate groups prevents the formation of noncovalent interactions between-PO_3_^−^ and the protonated amine groups of tn-2, as schematically presented in Figure 8. This molecular-level effect should be considered during analyses of the interactions between polyamines and nucleic acids because the interaction can influence the characteristics of gene expression.

According to the potentiometric and spectral results obtained for the Cu/CMP/tn-2 system, the Cu(CMP)H_2_(tn-2) complex is dominant at a pH of about 5. A comparison of the spectroscopic parameters of the complex, (*λ*_max_ = 717 nm and g_||_ = 2.231) with those of the Cu(CMP)H_2_(tn-1) species and those of earlier results obtained for similar systems [15, 16], points to the formation of a {1N,O_*x*_} chromophore. This conclusion is also confirmed by the similar values of the equilibrium constants for heteroligand complexes of tn-2 and tn-1, (log *K*_*e*_ = 5.98 and 5.97, resp.). Taking into regard the highly basic nature of tn-2, the pH range for formation of the complex suggests that the amine is fully protonated and the amine groups which are blocked from coordination are engaged in noncovalent interactions with the anchoring Cu(CMP) binary complex. The interaction mode is confirmed by the results of the NMR investigation. The changes in the positions of the NMR signals assigned to C(2), C(4), and C(5′) and the phosphorus atom of 0.278, 0.192, 0.243, and 0.279 ppm, respectively, indicate that the metal ion coordinates through the phosphate group and the endocyclic N(3) from the nucleotide. On the other hand, the changes in the chemical shifts assigned to C_1_, C_2_, and C_3_ from the amine of 0.079, 0.085, and 0.058 ppm, respectively, confirm the earlier postulated intermolecular interactions in which the protonated amine groups of tn-2 are involved as positive centres in noncovalent interactions with the phosphate group from the nucleotide (as a negative centre), despite the fact that this group is already involved in metal ion coordination. In view of the value of the negative charge on the potential interaction centres, the participation of the phosphate group is much more probable than the participation of the endocyclic N(3) atom. The values of the partial negative charges were obtained using the molecular electronic structure calculation program GAUSSIAN. The partial charges on the phosphate group and the N(3) atom of CMP are −1.179 and −0.709, respectively. A similar type of weak interaction was discussed above for the tn-1 system, in which the coordinated phosphate group simultaneously acts as the negative reaction centre.

In the ternary systems with tn-2, in contrast to those with tn-1, the nitrogen atoms from the polyamine are not involved in coordination. This is undoubtedly a result of steric hindrance provided by the two methyl groups in the tn-2 molecule.

## 4. Conclusions

The results of the potentiometric and spectral studies have shown that in the molecular complexes of 1,2*-*diaminopropane (tn-1) and 2-methyl-1,2-diaminopropane (tn-2) with nucleotides (NMP = AMP, CMP) the centres of noncovalent interactions are the phosphate groups of the nucleotides and the protonated amine groups of the polyamines. The differences in the interactions with the polyamines which act as models of biogenic amines are impacted by the presence of lateral chains (methylene groups) in tn-1 and tn-2. It should be emphasised that the Cu/NMP/tn-1 systems form complexes which are more stable than the complexes formed by the tn-2 systems. The tn-1 molecule has a single methyl group, while tn-2 has two methyl groups. In the ternary systems with metal ions, the heteroligand complexes are mainly the ML ⋯ L′ type, in which the protonated polyamine L′ is engaged in noncovalent interactions with the anchoring metal-nucleotide (ML) complex. The mode of coordination in the complex is mainly through the phosphate groups of the nucleotide with involvement of the endocyclic nitrogen atoms in a manner which depends upon the steric conditions and in particular on the number of the methylene groups in the polyamine molecule. The presence of the metal ion interferes substantially with the mode of interaction involved in formation of the amine-nucleotide adducts. For example, the copper (II) coordination with the phosphate group of the nucleotide, which is typical of metal/NMP systems, excludes the involvement of the –PO_3_^−^ group in the weak interactions (Figures 6 and 8). However, it should be noted that our results suggest the possibility of the phosphate groups being involved in noncovalent interactions with coordinated with metal ions. These types of interactions have been recently observed in similar systems with uridine-5*'*-monophosphate [46, 54].

## Figures and Tables

**Table 1:** Overall protonation constants, overall stability constants (log *β*), and equilibrium constants (log *K*_*e*_) of adducts formation in AMP/tn-1, AMP/tn-2, CMP/tn-1, and CMP)/tn-2 systems.

Systems	Equilibrium	log *β*	log *K*_e_*
Ligands	AMP + H^+^ ⇆ H(AMP)	6.43 (2)^[16]^	6.43
	AMP + 2H^+^ ⇆ H_2_(AMP)	10.45 (2)^[16]^	4.02
	CMP + H^+^ ⇆ H(CMP)	6.42 (2)^[16]^	6.42
	CMP + 2H^+^ ⇆ H_2_(CMP)	10.90 (2)^[16]^	4.48
	tn-1 + H^+^ ⇆ H(tn-1)	9.96 (1)	9.96
	tn-1 + 2H^+^ ⇆ H_2_(tn-1)	16.62 (1)	6.66
	tn-2 + H^+^ ⇆ H(tn-2)	9.80 (1)	9.80
	tn-2 + 2H^+^ ⇆ H_2_(tn-2)	16.41 (1)	6.61

AMP/tn-1	AMP + 4H^+^ + tn-1 ⇆ (AMP)H_4_(tn-1)	31.55 (6)	4.49
	AMP + 3H^+^ + tn-1 ⇆ (AMP)H_3_(tn-1)	27.63 (6)	4.58
	AMP + 2H^+^ + tn-1 ⇆ (AMP)H_2_(tn-1)	21.13 (6)	4.51
	AMP + H^+^ + tn-1 ⇆ (AMP)H(tn-1)	13.08 (6)	3.12

CMP/tn-1	CMP + 4H^+^ + tn-1 ⇆ (CMP)H_4_(tn-1)	30.87 (5)	3.91
	CMP + 3H^+^ + tn-1 ⇆ (CMP)H_3_(tn-1)	26.50 (4)	4.02
	CMP + 2H^+^ + tn-1 ⇆ (CMP)H_2_(tn-1)	20.20 (4)	4.14
	CMP + H^+^ + tn-1 ⇆ (CMP)H(tn-1)	12.50 (3)	2.91

AMP/tn-2	AMP + 4H^+^ + tn-2 ⇆ (AMP)H_4_(tn-2)	30.81 (5)	3.95
	AMP + 3H^+^ + tn-2 ⇆ (AMP)H_3_(tn-2)	26.76 (4)	3.92
	AMP + 2H^+^ + tn-2 ⇆ (AMP)H_2_(tn-2)	20.40 (4)	3.99
	AMP + H^+^ + tn-2 ⇆ (AMP)H(tn-2)	12.91 (3)	3.11

CMP/tn-2	CMP + 4H^+^ + tn-2 ⇆ (CMP)H_4_(tn-2)	30.77 (1)	3.46
	CMP + 3H^+^ + tn-2 ⇆ (CMP)H_3_(tn-2)	26.38 (1)	3.55
	CMP + 2H^+^ + tn-2 ⇆ (CMP)H_2_(tn-2)	20.19 (1)	3.78
	CMP + H^+^ + tn-2 ⇆ (CMP)H(tn-2)	12.88 (1)	3.08

*log *K*_e_ were calculated using protonation and overall stability constants of particular species, for example: log *K*_*e*(AMP)H3(tn-1)_ = log *β*_(AMP)H3(tn-1)_− log *β*_H(AMP)_− log *β*_H2(tn-1)_ = 27.63 − 6.43 − 16.62 = 4.58.

**Table 2:** Differences between ^13^C NMR and ^31^P NMR chemical shifts for the ligands in the AMP/tn-1, AMP)/tn-2, CMP/tn-1 and CMP/tn-2 systems in relation to the free ligands (ppm).

	AMP/tn-1 system										CMP/tn-1 system								
pH	AMP							tn-1			CMP						tn-1		
	C(2)	C(6)	C(5)	C(8)	C(4)	C(5′)	P_*α*_	C_1_	C_2_	C_3_	C(2)	C(4)	C(5)	C(6)	C(5′)	P_*α*_	C_1_	C_2_	C_3_

3	0.013	0.016	0.075	0.049	0.008	0.169	0.351	0.056	0.058	0.047	0.038	0.017	0.036	0.015	0.137	0.257	0.047	0.052	0.049
5	0.149	0.068	0.140	0.130	0.019	0.161	0.353	0.075	0.052	0.034	0.119	0.199	0.055	0.008	0.107	0.232	0.053	0.040	0.038
7	0.089	0.068	0.098	0.084	0.007	0.092	0.353	0.070	0.042	0.007	0.010	0.056	0.023	0.026	0.202	0.193	0.054	0.046	0.048
9	0.079	0.071	0.078	0.069	0.005	0.082	0.273	0.060	0.030	0.004	0.003	0.029	0.024	0.053	0.137	0.207	0.074	0.057	0.048

	AMP/tn-2 system										CMP/tn-2 system								
pH	AMP							tn-2			CMP						tn-2		
	C(2)	C(6)	C(5)	C(8)	C(4)	C(5′)	P_*α*_	C_1_	C_2_	C_3_	C(2)	C(4)	C(5)	C(6)	C(5′)	P_*α*_	C_1_	C_2_	C_3_

3	0.021	0.017	0.008	0.016	0.006	0.051	0.227	0.062	0.057	0.059	0.032	0.025	0.018	0.029	0.084	0.213	0.073	0.059	0.062
5	0.256	0.126	0.159	0.190	0.011	0.103	0.203	0.067	0.061	0.053	0.070	0.028	0.026	0.011	0.134	0.227	0.063	0.050	0.049
7	0.123	0.079	0.104	0.011	0.009	0.089	0.207	0.154	0.368	0.047	0.018	0.029	0.019	0.025	0.169	0.209	0.054	0.074	0.020
9	0.392	0.088	0.278	0.040	0.003	0.099	0.197	0.107	0.006	0.017	0.003	0.036	0.022	0.031	0.054	0.198	0.087	0.057	0.013

**Table 3:** Overall stability constants (log *β*) and equilibrium constants (log *K*_e_) of complexes formation in Cu(II)/tn-1^[49]^, Cu(II)/tn-2^[49]^, Cu(II)/AMP^[15]^, Cu(II)/CMP^[15]^, Cu(II)/AMP/tn-1, Cu(II)/AMP/tn-1, Cu(II)/CMP/tn-1, and Cu(II)/CMP/tn-2 systems.

Systems	Equilibrium	log *β*	log *K*_e_
Cu(II)/tn-1	Cu + H^+^ + tn-1 ⇆ CuH(tn-1)	15.48 (10)	5.52
	Cu + tn-1 ⇆ Cu(tn-1)	10.75 (7)	10.75
	Cu + 2(tn-1) ⇆ Cu(tn-1)_2_	19.60 (6)	8.85

Cu(II)/tn-2	Cu + H^+^ + tn-2 ⇆ CuH(tn-2)	14.51 (10)	4.71
	Cu + tn-2 ⇆ Cu(tn-2)	10.32 (2)	10.32
	Cu + 2(tn-2) ⇆ Cu(tn-2)_2_	19.45 (3)	9.13
	Cu + 3(tn-2) ⇆ Cu(tn-2)_3_	25.11 (5)	5.66
	Cu + 3(tn-2) + H_2_O ⇆ Cu(tn-2)_3_(OH) + H^+^	14.67 (6)	—

Cu(II)/AMP	Cu + AMP ⇆ Cu(AMP)	3.02 (8)	3.02
	Cu + AMP + H_2_O ⇆ Cu(AMP)(OH) + H^+^	−3.82 (5)	—

Cu(II)/CMP	Cu + CMP ⇆ Cu(CMP)	2.71 (6)	2.71
	Cu + CMP + H_2_O ⇆ Cu(CMP)(OH) + H^+^	−4.26 (8)	—

Cu(II)/AMP/tn-1	Cu + 3H^+^ + AMP + tn-1 ⇆ Cu(AMP)H_3_(tn-1)	30.73 (3)	—
	Cu + 2H^+^ + AMP + tn-1 ⇆ Cu(AMP)H_2_(tn-1)	26.03 (4)	6.39
	Cu + AMP + tn-1 ⇆ Cu(AMP)(tn-1)	14.86 (7)	11.84

Cu(II)/CMP/tn-1	Cu + 3H^+^ + CMP + tn-1 ⇆ Cu(CMP)H_3_(tn-1)	30.81 (1)	—
	Cu + 2H^+^ + CMP + tn-1 ⇆ Cu(CMP)H_2_(tn-1)	25.30 (6)	5.97
	Cu + CMP + tn-1 ⇆ Cu(CMP)(tn-1)	14.16 (2)	11.14

Cu(II)/AMP/tn-2	Cu + 3H^+^ + AMP + tn-2 ⇆ Cu(AMP)H_3_(tn-2)	29.63 (8)	—

Cu(II)/CMP/tn-2	Cu + 2H^+^ + CMP + tn-2 ⇆ Cu(CMP)H_2_(tn-2)	25.10 (4)	5.98


*log *K*_*e*_ were calculated using protonation and overall stability constants of particular species, for example: log *K*_*e* Cu(CMP)H2(tn-1) _ = log *β*_Cu(CMP)H2(tn-1)_ − log *β*_Cu(CMP)_ − log *β*_H2tn-1_ = 25.30 − 2.71 − 16.62 = 5.97.

**Table 4:** Visible and EPR spectral data for Cu(II)/AMP/tn-1, Cu(II)/AMP/tn-2, Cu(II)/CMP/tn-1, and Cu(II)/CMP/tn-2 systems.

Species	pH	*λ*_max_ (nm)	*g*_||_
Cu(AMP)H_3_(tn-1)	4.0	802	2.367
Cu(AMP)H_2_(tn-1)	5.0	697.5	2.243
Cu(AMP)(tn-1)	7.0	660	2.251
Cu(CMP)H_3_(tn-1)	4.0	769.5	2.332
Cu(CMP)H_2_(tn-1)	5.5	702	2.238
Cu(CMP)(tn-1)	7.0	633	2.263
Cu(AMP)H_3_(tn-2)	4.5	763	2.364
Cu(CMP)H_2_(tn-2)	5.0	717	2.231
